# The use of Latin terminology in medical case reports: quantitative, structural, and thematic analysis

**DOI:** 10.1186/s13256-018-1562-x

**Published:** 2018-02-23

**Authors:** Yuliia V. Lysanets, Olena M. Bieliaieva

**Affiliations:** Department of Foreign Languages with Latin and Medical Terminology, Ukrainian Medical Stomatological Academy, Poltava, Ukraine

**Keywords:** Medical discourse, Medical case report, Latin, Term, Terminological collocation

## Abstract

**Background:**

This paper focuses on the prevalence of Latin terms and terminological collocations in the issues of *Journal of Medical Case Reports* (February 2007–August 2017) and discusses the role of Latin terminology in the contemporary process of writing medical case reports.

**Methods:**

The objective of the research is to study the frequency of using Latin terminology in English-language medical case reports, thus providing relevant guidelines for medical professionals who deal with this genre and drawing their attention to the peculiarities of using Latin in case reports. The selected medical case reports are considered, using methods of quantitative examination and structural, narrative, and contextual analyses.

**Results:**

We developed structural and thematic typologies of Latin terms and expressions, and we conducted a quantitative analysis that enabled us to observe the tendencies in using these lexical units in medical case reports. The research revealed that the use of Latin fully complies with the communicative strategies of medical case reports as a genre. Owing to the fact that Latin medical lexis is internationally adopted and understood worldwide, it promotes the conciseness of medical case reports, as well as contributes to their narrative style and educational intentions.

**Conclusions:**

The adequate use of Latin terms in medical case reports is an essential prerequisite of effective sharing of one’s clinical findings with fellow researchers from all over the world. Therefore, it is highly important to draw students’ attention to Latin terms and expressions that are used in medical case reports most frequently. Hence, the analysis of structural, thematic, and contextual features of Latin terms in case reports should be an integral part of curricula at medical universities.

## Background

The profound influence of Latin upon the genesis and development of English medical terminology is undeniable and well-grounded [1–3]. Approximately 95% of English terms are borrowed from or created on the basis of Latin and latinized Greek [4]. Therefore, the English medical terminology cannot be “reasonably mastered without the knowledge of basic Latin” [5]. Within the English terminological system, Bujalkova and Dzuganova distinguish (1) Latin terms that were assimilated into English (anglicized Latin terms, such as “muscle,” “vein,” “nerve,” and so forth), (2) terms that experienced a multiple assimilation (from Greek into Latin, from Latin into Old French, from Old French into English; for instance, “spasmos” − “spasmus” − “spasme” − “spasm”), and (3) terms preserved in the original Latin form (“vena,” “dorsum,” “nucleus,” and so forth) [2]. The last-mentioned group of terms are preserved in the original Latin form up to the present but have undergone certain modifications in terms of pronunciation in accordance with English phonetic rules. Therefore, we suggest referring to this group of lexis as semiassimilated terms. In addition, we consider it necessary to distinguish the nonassimilated Latin terminology, represented by multiple-word terms that preserve the original features of the Latin grammatical system, such as the relationship between the parts of speech, agreement in gender, inflection rules, and so forth (for example, *per os*, *cor pulmonale*, *os ischii*, *musculus latissimus dorsi*, and the like).

Within the framework of medical discourse, virtually all genres are based largely on Latin and latinized Greek terminology. All medical research is built on the principles of *ab ovo* (“from the very beginning,” literally “from the egg”), *ab incunabulis* (literally “from the cradle”), and *ad fontes* (“back to the sources”), which provide the intergenerational continuity of medical science. Indeed, all areas of theoretical and practical medicine (biology, anatomy, physiology, pathology, pharmacology, clinical sciences, and so forth) as well as nomenclature corpora (taxonomies, International Nonproprietary Names, and so forth) are deeply rooted in the ancient nominative traditions. In this context, Latin occupies the firmest position in the anatomical vocabulary. Studies in Latin and Latin translations from Ancient Greek [6] “were the foundation of scientific thinking that was born in the boards of first universities” and “remain relevant in the current official anatomical terminology” [7]. *De humani corporis fabrica libri septem* (“On the Fabric of the Human Body in Seven Books,” 1543) by Andreas Vesalius not only contributed to the formation of anatomy as a separate academic subject [8] but also contained the data of pharmacological interest, such as the mention of *digitalis*, which is still used to treat heart failure. Pathology ranks second to the prevalence of Latin terminology; apart from Latin terms, clinical medicine displays more intensive expression of national languages [5].

Hence, it is natural that both original research (primary articles) and review articles (literature reviews, systematic reviews, and meta-analyses) use Latin terms extensively. Within the structure of primary research literature, it is also necessary to distinguish such an important genre as medical case report (MCR). In fact, MCRs are of interest because publications in this genre present a detailed report of diseases, the symptoms and their localization, management, and prognosis. That is, each MCR represents and integrates several medical domains and activities (description of anatomical structures and physiological conditions, laboratory studies and experiments, diagnosis and treatment, and so forth) at the same time. Therefore, it is relevant to determine to what extent the Latin terms are spread and how they are used in contemporary MCRs, as exemplified in the *Journal of Medical Case Reports* (*JMCR*). In such a way, our present research provides medical professionals with an appropriate terminological arsenal to be ready to deal with the genre of MCRs.

## Methods

The objective of the research is to study the frequency of using Latin terminology in English-language MCRs and, in such a way, to provide relevant guidelines for medical professionals to produce effective MCRs. The material used for the present research is the corpus of 1275 papers published in *JMCR* from February 2007 through August 2017. The material was selected by automatic search and sampling using the *Latin-Ukrainian Thesaurus of Clinical Terms* [9]. The search strategy was based on the authors’ teamwork: two groups of MCRs were formed and subsequently compared and discussed for their eligibility for inclusion. To standardize the selection of Latin terms, we developed the structural and thematic typologies of these units. The structural typology focuses on the major modeling patterns of Latin terminology in MCRs and comprises the following groups:

1. *One-word terms*: This category includes semiassimilated medical lexis that is deeply entrenched in the modern English language and is included in all English dictionaries (such as “abdomen,” “varicella,” “appendix,” and so forth). For objective reasons, these terms will not be subjected to quantitative analysis, because they virtually penetrate the entire English medical discourse. However, this group also embraces the Latin lexis, which is not that common and therefore can be studied quantitatively.
2. *Two-word phrases*: The group of two-word phrases is of particular interest because these terms are much less anglicized and preserve the original features of the Latin grammatical system. We believe that careful lexicogrammatical categorization of these terminological units will provide better understanding and deeper memorizing of them, which in turn will prevent possible spelling errors in MCR writing. We conducted quantitative analysis of the two-word terminological phrases and present them in descending order (highest to lowest frequency) within each subcategory. Hence, this group embraces the following subcategories:
  - Preposition + noun in ablative case
  - Preposition + noun in accusative case
  - Preposition + adjective in ablative case
  - Noun + adjective constructions
  - Other types of two-word phrases represented by miscellaneous constructions, such as noun + pronoun, preposition + pronoun, adverbial constructions, noun + noun in genitive case, noun + participle in genitive case, and verb + adverb
3. *Three-word phrases*: The group of three-word phrases comprises the following subcategories:
  - Noun + adjective + adjective
  - Noun + noun + adjective
  - Noun + adjective + noun
  - Noun + preposition + noun
  - Preposition + preposition + noun
4. A subgroup of compound English-Latin word phrases, which we refer to as the *hybrid terms*, containing both assimilated and nonassimilated lexical units
5. The group of abbreviations

Further, we organized the collected material into thematic groups and determined the frequency of their use in *JMCR*:

1. *Medical phenomena and processes*:
  - Anatomical descriptions
  - Physiological conditions
  - Methods of studies and experiments
  - Indications for treatment and routes of administering medications
  - Pathological conditions
2. *Academic language*

The selected MCRs were considered using the method of lexicogrammatical and stylistic analyses, with a focus largely on the structural peculiarities, narrative function, and contextual role of Latin terminology. The use of Latin terms and phrases was subjected to quantitative examination to determine their frequency.

## Results

We studied 1275 papers published in *JMCR* from February 2007 through August 2017 and containing Latin terms and terminological phrases. Within the structure of the selected research material, MCRs constitute an overwhelming majority (*n* = 1232), followed by errata (*n* = 17), research articles (*n* = 14), reviews (*n* = 10), and editorials (*n* = 2). We developed the structural and thematic typologies of Latin terms and conducted quantitative analysis of these terms. In this way, we determined the frequency of using Latin in MCRs, as well as detected the most prevalent and productive lexical units and phrases. The structural typology comprises the following groups:

1. *One-word terms*: We found four MCRs with the adverb *mane* (meaning *in the morning*), which is used in prescriptions, for example: “She was treated for MDD with paroxetine 20 mg/*mane* in 2002” [10]. There are four publications with the preposition *circa* (meaning *approximately* or *about*), which is used for descriptive purposes, for instance: “a reduced debit of the fistula of *circa* 10 mL” [11]. We detected 17 cases of using the noun *erratum* (meaning *error*) for amending a published text. Furthermore, the one-word terms are represented by compound Latin lexis, such as *primigravida* (“a woman who is pregnant for the first time”; *n* = 30 cases) and *nullipara* (“a woman who has never given birth”; *n* = 2 cases).

In this context, it is necessary to remark that pluralizing one-word Latin terms can sometimes be quite a challenge. For instance, a common mistake occurs when deriving the plural form of the Latin word *septum*. This lexical unit belongs to the second declension of Latin nouns, neuter gender. Therefore, the correct plural form in Latin (and in English) is *septa*. However, the plural form *septa* is quite often mistaken for a singular form and consequently is erroneously pluralized as *septae* (on the model of *vertebra*/*vertebrae*: the first declension, feminine gender). As a result, a misspelling (*septae*) occurs. In fact, we found 66 publications in *JMCR* with the correct plural form of the word under consideration. At the same time, it is to be noted that 20 papers contain the incorrect plural form of this word, for example: “In non-fibrotic lung tissue the alveolar *septa* were edematous with sloughing of pneumocytes” [12]; “surgical drainage of the hepatic abscess (that contained many *septa*) was performed” [13]; “It also facilitates the removal of debris, purulent secretions, *septa*” [14]; “Alveolar *septa* were inflamed, thickened and fibrotic” [15]; “eosinophilic cytoplasm delineated by thin fibrovascular *septa* which separated the tumor cells” [16]; “a 14.7 × 13.6 × 15.2 cm complex septated cystic mass arising from the peritoneum, containing enhancing *septa* and solid nodules” [17].

A similar error may occur with the word *dorsum*, which also belongs to the second declension of Latin nouns, neuter gender. We found three cases of using the correct plural form of this word (*dorsa*) and one MCR with a misspelling (*dorsae*): “Her dermographism was improving but she had developed confluent erythema and slight hyperkeratosis between and over the *dorsa* of her fingers” [18].

Another challenging aspect of using Latin in MCRs is the subject-verb agreement in number. This type of error is not widespread within the selected research material, and only isolated cases were detected with the words *bacterium* (singular)/*bacteria* (plural) and *labium* (singular)/*labia* (plural): “The next closest *bacterium* was *H. parainfluenzae* with a 97% similarity score” [19]; “[r]ight *labium* was asymmetrically enlarged” [20].

Thus, we believe that it is essential to draw authors’ attention to such potential errors when using Latin terms to improve the quality of MCRs writing.

2. The group of two-word phrases embraces the following subcategories:
  - Preposition + noun in ablative case: *in vitro* (literally “in the glass,” meaning “performed outside the normal biological context”; *n* = 192 cases); *in situ* (“on site”; *n* = 191 cases); *in utero* (“in the womb”; *n* = 44 cases); *ex utero* (“outside the uterus”; *n* = 4 cases); *in silico* (meaning “performed on computer or via computer simulation”; *n* = 3 cases); *ex vitro* (literally “outside the glass” or outside the artificial tissue; *n* = 2 cases); *ab initio* (“from the beginning”; *n* = 1 case); and *de facto* (“in fact”; *n* = 1 case).
  - Preposition + noun in accusative case: *per rectum* (“by way of the rectum”; *n* = 70 cases); *postpartum* (“after birth”; *n* = 41 cases); *per os* (“by mouth”; *n* = 30 cases); *in toto* (“in general”; *n* = 6 cases); *ante mortem* (“before death”; *n* = 5 cases); *ante partum* (“before birth”; *n* = 1 case); *ad hoc* (“for this”; *n* = 1 case); *inter alia* (“among other things”; *n* = 1 case); and *post factum* (“after the event”; *n* = 1 case).
  - Preposition + adjective in ablative case: *in vivo* (literally “within the living,” meaning tested in the living organism; *n* = 71 cases); *ex vivo* (“out of the living,” referring to something that takes place outside a living organism; *n* = 5 cases); *a posteriori* (literally “from what comes after,” meaning when justification is dependent on experience; *n* = 3 cases); *a priori* (literally “from the earlier,” meaning when justification is independent of experience; *n* = 2 cases); and *ex novo* (“from the beginning”; *n* = 2 cases).
  - Noun + adjective constructions are represented by the following examples: *labia majora* (“outer vulvar lips”; *n* = 16 cases); *placenta previa* (an obstetric complication in which the placenta is inserted partially or wholly in the lower uterine segment; *n* = 12 cases); *foramen magnum* (“great foramen”; *n* = 10 publications); *cor pulmonale* (“pulmonary heart”; *n* = 10 cases); *vastus medialis* (“medial vastus muscle”; *n* = 10 cases); *vastus lateralis* (“lateral vastus muscle”; *n* = 8 cases); *placenta accreta* (an obstetric complication in which the chorionic villi are attached to the myometrium; *n* = 5 cases); *oculus dexter* (“the right eye”; *n* = 4 cases); *oculus sinister* (“the left eye”; *n* = 4 publications); *coxa vara* (“a deformity of the hip”; *n* = 4 cases); *placenta increta* (an obstetric complication in which the chorionic villi penetrate the myometrium; *n* = 3 cases); *virgo intacta* (“a virgin”; *n* = 2 cases); *os sacrum* (“sacral bone”; *n* = 2 publications); *os ilium* (“iliac bone”; *n* = 2 cases); *placenta percreta* (chorionic villi penetrate the perimetrium; *n* = 2 cases); *genu valgum* (“the knock-knee deformity”; *n* = 2 cases); *hallux valgus* (“the skewed toe”; *n* = 1 case); *os peroneum* (“peroneal bone”; *n* = 1 case); *os odontoideum* (“odontoid bone”; *n* = 1 case); *os trigonum* (“triangular bone”; *n* = 1 case); *musculus sartorius* (“tailor’s muscle”; *n* = 1 case); and *labia minora* (“inner vulvar lips”; *n* = 1 case).
  - Other types of two-word phrases are not so widespread in the analyzed MCRs as in the previous groups, and this subcategory is represented by miscellaneous constructions, such as noun + pronoun: *status quo* (literally “the state in which,” meaning “the state of things”; *n* = 2 cases); preposition + pronoun: *per se* (“by itself”; *n* = 27 cases; and adverbial constructions: *sensu stricto* (“in the narrow sense”; *n* = 2 cases); *sensu lato* (“in the broad sense”; *n* = 1 case); noun + noun in genitive case: *caput Medusae os* (“the radiating appearance of the superficial epigastric veins”; *n* = 2 cases); *pubis* (“bone of pubis”); *os calcis* (“bone of heel”); and *os ischii* (“ischial bone”) (*n* = 1 case for each unit); and noun + participle in genitive case: *modus operandi* (“mode of operation”; *n* = 1 case); verb + adverb: *vide supra* (“see above”; *n* = 1 MCR).
3. The group of three-word phrases is also remarkable for preserving the original features of Latin grammar. It comprises the following subcategories:
  - Noun + adjective + adjective: *musculus rectus abdominis* (“abdominal rectus muscle”), *musculus gastrocnemius medialis* (“medial calf muscle”), *os tibiale externum* (“external tibial bone”), *ductus hepaticus dexter* (“right hepatic duct”), and *placenta previa percreta* (an obstetric complication in which the chorionic villi penetrate the perimetrium) (*n* = 1 case for each unit).
  - Noun + noun + adjective: *abductor digiti minimi* (“little finger muscle”; *n* = 6 cases); *levator palpebrae superioris* (“the muscle that elevates the upper eyelid”; *n* = 3 cases); *abductor pollicis brevis* (“the short abductor muscle of thumb”; *n* = 3 cases); *abductor pollicis longus* (“the long abductor muscle of thumb”; *n* = 2 cases); *levator labii superioris* (“the lifter of the upper lip”; *n* = 1 case); and *locus resistentiae minoris* (“an area of little resistance”; *n* = 1 case).
  - Noun + adjective + noun: *musculus orbicularis oculi* (“orbicular muscle of eye”), *musculus latissimus dorsi* (“broadest muscle of back”), and *musculus rectus abdominis* (“abdominal rectus muscle”) (*n* = 1 case for each unit).
  - Noun + preposition + noun: *carcinoma in situ* (“a group of abnormal cells, located in the place where they first formed”; *n* = 26 cases) and *fissula ante fenestram* (“a small connective tissue-filled cleft, located anterior to the oval window”; *n* = 1 publication).
  - Preposition + preposition + noun: *ex post facto* (“from a thing done afterward”; *n* = 1 case). It should be remarked that the latter construction is rather unusual for Latin; yet, such a rare expression still can be found in current medical discourse.

Noteworthy is the contextual use of the expression *in situ*. As is evident from the foregoing, this phrase can be used in oncology (*carcinoma in situ*), as well as in the process of measurement taking, viewing anatomical structures, and medical simulation (for example, “She was treated operatively with an *in situ* cannulated hip screw fixation and healed completely within eight weeks” [21]), depending on the context. Hence, this is one of the most productive Latin lexical units in modern medical discourse.

2. Further, we distinguished a subgroup of compound English-Latin word phrases (we refer to them as the *hybrid terms*) containing both assimilated and nonassimilated lexical units: *inferior vena cava* (*n* = 122 cases), *latissimus dorsi muscle* (*n* = 6), *latissimus dorsi flap* (*n* = 6), *carcinoma ex pleomorphic adenoma* (*n* = 5), *dorsum of the tongue* (*n* = 5), *the abductor hallucis muscle* (*n* = 4), *quadriceps femoris muscle* (*n* = 3), *levator palpebrae muscle* (*n* = 3), *orbicularis oris muscle* (*n* = 2), *the rectus femoris muscle* (*n* = 2), *ex vacuo dilatation* (*n* = 1), and *quadratus femoris* muscle (*n* = 1).
3. The group of abbreviations is represented by lexical units such as *i.e.* (*id est*, meaning “that is”; *n* = 85 cases); *e.g.* (*exempli gratia*, meaning “for example”; *n* = 81 cases); *etc.* (*et cetera*, meaning “and so on”; *n* = 18 cases); *q.i.d.* (*quarter in die*, meaning “four times per day” as used in prescriptions; *n* = 12); *b.i.d.* (*bis in die*, meaning “twice per day”; *n* = 11); *t.i.d.* (*ter in die*, meaning “three times per day”; *n* = 8); *PRN* (*pro re nata*, meaning “as and when necessary”; *n* = 1); *qAM* (*quaque die ante meridiem*, meaning “every morning”; *n* = 1); *qHS* (*quaque hora somni*, meaning “every bedtime”; *n* = 1); and *qPM* (*quaque die post meridiem*, meaning “every evening”; *n* = 1).

As a next step, we organized the collected material into thematic groups and determined the frequency of their use in *JMCR*. Hence, the thematic typology comprises the groups of Latin terms that signify the following:

1. Medical phenomena and processes:
  - Anatomical descriptions (for example, phrases with terms such as *musculus, os*, *levator*, *abductor*, and so forth)
  - Physiological conditions (for example, *virgo intacta*, *primigravida*, *nullipara*, *ante partum*, *postpartum*, and so forth)
  - Methods of studies and experiments (for example, *in vivo*, *in vitro*, *in situ*, and so forth)
  - Indications to treatment and routes of administering medications (for example, *mane*, *ter in die*, *quarter in die*, *per os*, and so forth)
  - Pathological conditions (for example, *cor pulmonale*, *carcinoma ex pleomorphic adenoma*, *placenta previa percreta*, and so forth)
2. Academic language that maintains the coherence and cohesion of the discourse (for example, *inter alia*, *in toto*, and so forth).

Table 1 demonstrates the results of our quantitative analysis and shows the correlation between the structure and thematic content of Latin terms in *JMCR.*

**Table 1:** Latin terms and terminological collocations in *Journal of Medical Case Reports*

Thematic groups	Anatomical descriptions	Physiological conditions	Methods of studies and experiments	Indications to treatment	Pathological conditions	Academic language
Structure						
One-word terms	N/A^a^	32	–	4	N/A*	21
Two-word phrases	64	97	464	100	33	52
Three-word phrases	24	–	–	–	27	1
Compound English-Latin word phrases	151	6	–	–	5	–
Abbreviations	–	–	–	35	–	184

^a^ We did not conduct quantitative analysis of one-word Latin terms denoting anatomical descriptions and pathological conditions because of their predominance in the medical discourse and because of their high level of assimilation into the English language. Instead, we deliberately focused on the multiple-word terms that clearly preserve the Latin lexicogrammatical features and therefore are the demonstrative examples of using the classical language in MCRs nowadays

*N/A* Not available, *MCRs* Medical case reports

## Discussion

The research revealed that Latin terminology is most frequently used to refer to methods of studies and experiments (464 two-word phrases). Next are the categories of anatomical descriptions (236) and academic language (258), followed by indications for treatment (139 units). Latin terminology, used for denoting physiological conditions, is represented by 135 units, whereas the group of pathological conditions is the least “latinized” category (65 cases). In fact, the majority of MCRs under consideration give preference to English equivalents of names of pathological conditions (for instance, *acute abdomen* rather than *abdomen acutum* as in [13]).

In our opinion, Latin terminology is well-preserved in the names for methods of studies and experiments, because it is neutral (favoring no particular national language), constant, and internationally accepted. Furthermore, it is necessary to observe that this group of terms is dominated by two-word expressions, which enable the authors to render their message accurately and concisely.

One cannot overestimate the role of Latin in anatomical nomenclature, “whose international version remains Latin in the full extent” [5]. Among the advantages of Latin in anatomy, one should mention terminological continuity and constancy [5], which date back to early human history. In fact, the first anatomical descriptions were written by Hippocrates, Aristotle, and Galen and subsequently improved by Vesalius, Fabricius, and Harvey [22]. For instance, the names of muscles have not changed since their introduction by Vesalius in 1543 [23]. Thus, Latin anatomical terminology provides a rich and well-established stock of vocabulary that releases modern researchers from the necessity of “reinventing the wheel.”

Moreover, it is highly important to use Latin correctly. Indeed, the use of correct Latin terms reflects the author’s excellence and scholarly accomplishments. It is also essential for “the rigor, stability, and universality of the nomenclature” [24]. The problems of correct use of Latin terms in medical research have repeatedly attracted scholars’ attention. For instance, Neumann has extensively studied these issues in anatomy [24–28]. It is necessary to instruct students about the potential spelling errors that may occur when using Latin terminology, for example, in the subject-verb agreement in number or when pluralizing terms such as *septum*, *dorsum*, *labium*, and so forth. Table 2 demonstrates the most challenging cases of using plural forms of Latin in MCRs.

**Table 2:** Latin plural endings in English medical terminology

Singular	Plural	Singular	Plural
Vertebra	Vertebrae	Bacillus	Bacilli
Atrium	Atria	Bronchus	Bronchi
Bacterium	Bacteria	Focus	Foci
Curriculum	Curricula	Fungus	Fungi
Datum	Data	Nucleus	Nuclei
Dorsum	Dorsa	Stimulus	Stimuli
Erratum	Errata	Analysis	Analyses
Labium	Labia	Apex	Apices
Medium	Media	Appendix	Appendices
Septum	Septa	Axis	Axes
Stratum	Strata	Crisis	Crises
Symposium	Symposia	Diagnosis	Diagnoses
Criterion	Criteria	Index	Indices
Ganglion	Ganglia	Paralysis	Paralyses
Phenomenon	Phenomena	Thesis	Theses

To facilitate memorization, Latin terms are grouped on the basis of their declension and gender

Latin terms in the names of indications for treatment (such as *PRN* [*pro re nata*], *t.i.d.* [*ter in die*], and so forth) are widely used in the modern clinical settings all over the world. This is due to the fact that these terms are transnational, stable, and internationally understood. Furthermore, such terms are effective and concise tools of conveying the author’s message as quickly as possible.

Latin has a unique status as the fundamental principle of scientific style and academic language. Mastering the basics of Latin has survived from ancient times as a continuous tradition and an effective means of capturing, reflecting, and disseminating scholarly knowledge. In fact, scientific genres such as thesis, monograph, article, report, polemic presentation, and textbook were written in Latin until as late as the 19th century. Unlike many ancient languages that are now forgotten, Latin became the language of science with a clearly focused international communicative status, particularly in medicine, and “went far beyond the territory occupied by its speakers in ancient times” [8]. Throughout human history, Latin performed the epistemological function and served as a means of “accumulation, reception, transmission and popularization of achievements in various areas of medical science” [8]. Nowadays, the use of Latin academic expressions is a strong tradition in scholarly communities. In other words, Latin maintains the position of *lingua franca* among educated people*,* regardless of their nationality. Besides this respect for tradition, these terms enable authors to express themselves succinctly and concisely.

The pathophysiology nomenclature is rooted in the ancient Greek tradition, which subsequently underwent latinization. For instance, terms such as *arthritis*, *carcinoma*, *cholera*, *emphysema*, *erythema*, *herpes*, *kyphosis*, *nephritis*, *pleuritis*, *typhus*, and many others were first described in *Corpus Hippocraticum* (in the fifth to fourth centuries b.c.e.) and are still used today. Among the names for pathological conditions, one should also mention eponyms, such as *facies Hippocratĭca*, *succussio Hippocrătis*, *unguis Hippocratĭcus*, and so forth, which are also internationally used and understood. In our opinion, such eponymic terms disclose the evolution of medical research and practice, provide continuity of scientific knowledge, and contribute to the formation of terminological competence of medical students [29]. Furthermore, eponyms “bring colour to medicine, embed medical traditions and culture to our history” [30]. Thus, Latin clinical terminology is the result of the centuries-old history of world medical development, which provides a neutral ground for medical professionals from different countries. Moreover, Latin and latinized Greek are productive tools for coining new terms. For instance, the term *appendicitis* was used for the first time in 1886 in *American Journal of Medical Science*. Hence, the ability to build new compound words also indicates the viability of this terminological corpus.

In terms of structure, one-word terminological phrases (that is, pure Latin terms) are found in 57 MCRs (physiological conditions, *n* = 32; academic language, *n* = 21; indications for treatment, *n* = 4). At the same time, the one-word semiassimilated terms were not calculated in this study, owing to their widespread occurrence in the medical language and strong penetration into English dictionaries. Meanwhile, the nonassimilated multiple-word terms are of particular interest because this lexical layer preserves the grammatical features of Latin. The two-word terms are used most often, comprising 810 cases (mostly the methods of studies and examination, *n* = 464; followed by indications for treatment, *n* = 100; physiological conditions, *n* = 97; anatomical descriptions, *n* = 64; academic language, *n* = 52; and pathological conditions, *n* = 33). Three-word collocations are least frequently used, comprising 52 cases (anatomical descriptions, *n* = 24; pathological conditions, *n* = 27; and academic language, *n* = 1); compound (“hybrid”) English-Latin word phrases, comprising 162 cases (mostly anatomical descriptions, *n* = 151; followed by physiological conditions, *n* = 6; and pathological conditions, *n* = 5); and abbreviations, comprising 219 cases (academic language, *n* = 184; indications to treatment, *n* = 35).

The present study demonstrates the long-standing predominance and viability of Latin in modern MCRs. One can observe that the undeniable advantage of one-word Latin terms, as in the example of *mane*, is their conciseness, which is essential for MCRs as a genre [31]. Likewise, two-word Latin terms, such as *in vitro*, *ex vitro*, *in vivo*, *ex vivo*, *in situ*, and so forth, as well as abbreviations enable the transfer of the maximum amount of necessary information using the minimum linguistic tools.

In modern MCRs, three-word Latin terms, for example, *abductor digiti minimi*, *levator palpebrae superioris*, and so forth, are represented largely by anatomical notions. This feature results from the well-developed system of anatomical terminology that was meticulously arranged and organized by the ancient Romans. Hence, it is essential for medical professionals to be aware of correct forms of nominative plurals, genitive singular and plural, Latin adjectives, and so forth in order to avoid misspelling in medical writing.

The use of “hybrid” (English-Latin) word phrases in MCRs indicates that Latin and English in modern medical terminology reside in a state of natural symbiosis. We strongly agree with Marecková *et al*. [5] that, on the one hand, Latin has found its “continuation” in English and thus has managed to retain “its unique standing” and, on the other hand, the spread of English medical terminology all over the world was facilitated largely by its Latin origins.

Several studies [5, 6, 8] have addressed the issue of replacement of Latin by English. It is our opinion that, despite the spread of the English language, Latin firmly maintains its important and relevant position in the modern world. First, Latin is unbound in terms of territory: It possesses a unique status of a globally neutral vehicle of medical communication, and thus its use gives respect to other national languages. It should be remembered that the official languages of the United Nations (UN) are the six languages used for written documents and meetings of the UN. In other words, Latin terms are not only international but even “supranational.” Latin terminology is unambiguous, succinct, concise, and easy to pronounce. Furthermore, knowledge of Lain etymologies promotes correct English spelling and understanding of medical terminology. Moreover, through studying Latin, a person gains an understanding of the mechanisms and structure of any other language [32]. In addition, for representatives of Slavic nations, Latin becomes virtually a key to Western civilization because it is incorporated into most European languages. Hence, by means of learning Latin, English medical terminology becomes transparent and understandable for the Slavs. It should be noted that most Latin and latinized Greek terms have been used for over 2000 years. As a result, the use of Latin provides the intellectual and terminological continuity of Western medicine that is rooted in ancient times. It is also necessary to bear in mind that the original version of *Magna Charta Universitatum Europaeum* (1986) was written in Latin to celebrate and encourage the deepest values of European university traditions.

Thus, using Latin terms promotes the conciseness of MCRs, contributes to the coherence and cohesion of narratives in MCRs, and constitutes a rich and viable academic layer of medical terminology. Thus, our present research reveals that the use of Latin fully complies with the communicative strategies of MCRs as a genre, such as conciseness, narrative style, and educational value [31].

It is also essential to identify the countries where Latin terminological units are the most common in medical writing. According to our country-wise analysis of using Latin in MCRs, the following are the countries where Latin terminological units are the most common in medical writing (from highest to lowest frequency): the United Kingdom, the United States, Italy, India, Germany, Australia, Greece, France, Japan, Malaysia, China, Nigeria, Netherlands, Belgium, Finland, Argentina, Portugal, Spain, Turkey, Romania, Poland, Kosovo, New Zealand, Senegal, Tanzania, Cameroon, Egypt, Iran, Brunei, and Thailand.

Recent research [33] has been focused on the relevance of Latin in the modern curricula at universities. Marecková *et al*. referred to the neo-Latin adage “*Invia est in medicina via sine lingua Latina*” (“The way without Latin is impassable in medicine”) [5] and advocated the need for teaching Latin terminology at medical universities. These scholars contended that it is essential to provide students “with a functional instruction on precise and linguistically correct usage of the terminological apparatus” [5]. Rein conducted a survey of students in Estonia and found that more than half of respondents answered that Latin continues to maintain its position “as the international language of medicine” [33]. Smith *et al*. [34] provided scientific evidence that students’ awareness of Greek and Latin etymologies improves their academic performance when learning medical terminology. Turmezei [35] asserted that understanding anatomical etymology enhances the training process and reinforces memorization. Likewise, Stephens and Moxham [36] contended that medical students should acquire basic understanding of and “have some formal or informal instruction in classical Greek and Latin” as they pertain to medical terminology. In our previous research [37, 38], we also observed that instruction in medical terminology promotes the development of core competencies of a future doctor.

It is our belief that the analysis of structural, thematic, and contextual features of Latin terms in case reports should be an integral part of curricula at medical universities. In countries where higher medical education includes academic subjects such as “Latin and Medical Terminology” and/or “English for Specific Purposes” (for example, in Ukraine, Bulgaria, and Russia), the use of Latin in MCRs should be added as an extracurricular submodule during the first year of study. At the same time, for countries where Latin and English are not a compulsory part of the medical curriculum, these issues should be discussed in the framework of an additional module (for example, “Latin for Academic Purposes”).

The results of this research will be integrated into the curriculum of Ukrainian Medical Stomatological Academy with the aim of improving the writing proficiency among undergraduate students. The implementation plan comprises the following steps:

1. Curriculum planning and design
  - Defining curriculum objectives and instructional strategies
  - Identifying the stakeholders
  - Analyzing students’ needs and staff resources
  - Reviewing the existing curricula and available instruction resources
  - Developing and expanding the cocurricular resources: republication of educational literature, developed by the authors of this paper (*Latin-Ukrainian Thesaurus of Clinical Terms* [9] and *English for Professional Use: Dentistry* [39]), with additional units and sections on Latin terms and expressions that are used in MCRs most frequently; publication of *Medical English for Academic and Teaching Purposes* [40], focused on improvement of the writing skills demonstrated in MCRs and development of teaching staff
  - Aligning the submodule with the existing curricula of academic subjects “Latin and Medical Terminology” and “English for Specific Purposes” that Ukrainian students learn during the first and second years of study
2. Project management
  - Integrating the results of the present research with the existing curriculum in Ukraine through adding an extracurricular submodule on “Latin for Academic Purposes” to medical training courses
  - Monitoring the project by analyzing short-term, midterm, and end-of-year outcomes (collecting samples of student writing to measure progress)
  - Quality control by questionnaire surveys of students
  - Reviewing the results and troubleshooting
  - Evaluating and communicating the results with the stakeholders
3. Incorporating best practices by developing comprehensive national guidelines for using Latin in MCRs for further dissemination

## Conclusions

The adequate use of Latin terms in MCRs is an essential prerequisite of effective sharing one’s clinical findings with fellow researchers from all over the world. First, using Latin promotes the conciseness of MCRs because the Latin lexis is internationally adopted and understood. Second, Latin expressions contribute to the coherence of narratives in MCRs. Third, Latin terms constitute an ever-present and timeless lexical layer of medical terminology, and their appropriate use adds to the overall scholarly value and educational intentions of MCRs. Therefore, it is highly important to draw the attention of future medical professionals to the Latin terms and expressions that are used in case reports most frequently. It is our belief that the use of Latin in MCRs requires further examination in other scholarly journals that are registered in PubMed and other databases [41–44], which will consequently enable the conduct of comparative studies of different academic periodicals and provide a deeper understanding of the role of Latin in modern medical discourse.

## Abbreviations

*JMCR*: *Journal of Medical Case Reports*
MCR: Medical case report
UN: United Nations
